# Correlating Glycoforms of DC‐SIGN with Stability Using a Combination of Enzymatic Digestion and Ion Mobility Mass Spectrometry†

**DOI:** 10.1002/anie.202005727

**Published:** 2020-07-07

**Authors:** Hsin‐Yung Yen, Idlir Liko, Joseph Gault, Di Wu, Weston B. Struwe, Carol V. Robinson

**Affiliations:** ^1^ Physical and Theoretical Chemistry Department of Chemistry University of Oxford South Parks Road Oxford OX1 3TA UK; ^2^ Present address: OMass Therapeutics The Schrodinger Building, Oxford Science Park Oxford OX4 4GE UK

**Keywords:** DC-SIGN, glycoprotein, intact mass spectrometry, ion mobility, O-glycosylation

## Abstract

The immune scavenger protein DC‐SIGN interacts with glycosylated proteins and has a putative role in facilitating viral infection. How these recognition events take place with different viruses is not clear and the effects of glycosylation on the folding and stability of DC‐SIGN have not been reported. Herein, we report the development and application of a mass‐spectrometry‐based approach to both uncover and characterise the effects of O‐glycans on the stability of DC‐SIGN. We first quantify the Core 1 and 2 O‐glycan structures on the carbohydrate recognition and extracellular domains of the protein using sequential exoglycosidase sequencing. Using ion mobility mass spectrometry, we show how specific O‐glycans, and/or single monosaccharide substitutions, alter both the overall collision cross section and the gas‐phase stability of the DC‐SIGN isoforms. We find that rather than the mass or length of glycoprotein modifications, the stability of DC‐SIGN is better correlated with the number of glycosylation sites.

## Graphical abstract

**Uncovering stability of DC‐SIGN glycoforms with native mass spectrometry**: A combined strategy of exoglycosidase sequencing, native mass spectrometry, ion mobility, and gas‐phase unfolding uncovered the occupancy and structural detail of O‐glycans present on the carbohydrate binding domain of DC‐SIGN. Collision‐induced unfolding by ion mobility revealed differences in stability among glycoforms, independent of glycoprotein mass.

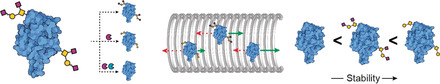

While much current interest focuses on the glycosylation status of the viral spike protein of SARS‐CoV‐2, few studies have addressed the role of receptor glycosylation. DC‐SIGN (the innate immune receptor dendritic cell‐specific intercellular adhesion molecule‐3 grabbing non‐integrin) has been implicated as an “infection enhancer” in previous reports of coronavirus epidemics.^1^, ^2^ This property is attributed to the ability of DC‐SIGN either to recognize self‐ or other pathogenic carbohydrates. As such DC‐SIGN is proposed to play an unfavorable role in coronavirus infections, enhancing circulation of virions through multivalent interactions of high‐mannose type viral glycans via its carbohydrate recognition domain (CRD).^3^ This ability to increase circulation of viral particles has prompted efforts to develop glycomimetic drugs for DC‐SIGN, as CRD antagonists, to inhibit host–virus interactions and infection.^4^ To our knowledge however, DC‐SIGN, a C‐type lectin expressed on the surface of dendritic cells and macrophages, has yet to be reported as O‐glycosylated and the extent and heterogeneity of these glycoforms has yet to be defined.

Here we use DC‐SIGN as a challenging test case to develop and apply high‐resolution native mass spectrometry (MS) and ion mobility (IM) instrumentation to study the effects of glycosylation on the biologically relevant forms of the receptor. DC‐SIGN is a type II membrane protein comprising three main domains: a cytoplasmic region, a transmembrane segment, and an extracellular domain (ECD). Although DC‐SIGN is known to be tetrameric, enabling multivalent interaction with pathogens, a complete structure is not available; largely due to the intrinsic flexibility of the ECD. The ECD can be divided into two distinct regions: a neck region involved in tetramerization of the receptor and CRD, which mediates the molecular recognition processes. A model of the ECD has been proposed, based on small angle X‐ray scattering data.^5^

Our principal goal is to understand how specific glycans impact the stability of the DC‐SIGN receptor by developing and applying MS approaches. Delineating glycan‐specific attributes is challenging because of glycoprotein diversity and limitations in structural or biophysical methods capable of interrogating single protein glycoforms. MS could potentially address challenges arising from glycoprotein heterogeneity, which are described by variations in glycosite occupancy (macroheterogeneity) and diversity among glycan structures at a single glycosylation site (microheterogeneity). These two types of data are generally obtained separately through orthogonal MS and liquid chromatography techniques. Herein, we show that both macro‐ and microheterogeneity information can be acquired simultaneously through a single MS experiment using specific monosaccharide glycosidases in sequence. We further show, using IM and collision‐induced unfolding (CIU) measurements, that specific glycan structures, as well as the extent of their occupancy, affect the stability of the intact glycoprotein. Intriguingly, we observed a correlation between gas‐phase stability of DC‐SIGN and specific glycan features, as opposed to a direct connection between glycoprotein mass and unfolding. Taken together this approach therefore provides a means to gain both structural and biophysical information for this intact folded glycoprotein that is not accessible by other static or ensemble‐based methods.

We began our investigation by expressing and purifying DC‐SIGN CRD from human embryonic kidney (293T) cells. The native MS spectrum of this protein revealed two major charge states (8^+^ and 9^+^) with seven clear proteoforms within each distribution (Figure 1 a). The theoretical mass of non‐glycosylated DC‐SIGN CRD is 19 128 Da. The lowest mass observed was 21 014.5±0.4 Da with additional peaks indicating the presence of further post‐translational modifications (PTMs). The mass differences between these peaks correspond to monosaccharides with distinct numbers of hexose (+162 Da), *N*‐acetylhexosamine (+203 Da), and *N*‐acetylneuraminic acid (+292 Da) residues. The difference of +1886.5±0.4 Da is consistent with two hexoses (Hex), two *N*‐acetylhexosamines (HexNAc), and four *N*‐acetylneuraminic acid residues (*N*‐acetylneuraminic acid, Neu5Ac, is referred to as sialic acid herein). Further mass shifts on the CRD domain are from Hex‐HexNAc disaccharide additions plus a further two sialic acids. At this stage of the analysis HexNAc monosaccharides are unspecified (blue/yellow squares) as they can be either *N*‐acetylgalactosamine and/or *N*‐acetylglucosamine residues. We then recorded the native mass spectrum of the full ECD domain and observed a closely similar glycosylation pattern (Figure 1 b). We conclude that the CRD/ECD glycan modifications range between (Hex‐HexNAc)_2–6_ with either four or six sialic acids—the most glycosylated forms having six Hex, six HexNAc, and six Neu5Ac monosaccharides (Figure 2 a).

**Figure 1:**
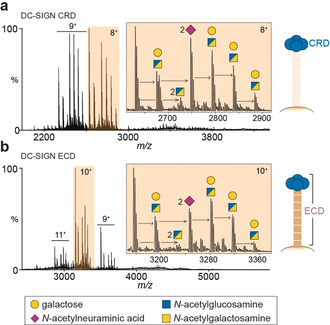
Identification of O‐glycosylation on DC‐SIGN. a) Native mass spectrum of the DC‐SIGN carbohydrate recognition domain (CRD) monomer displaying two major charge states (8^+^ and 9^+^). Peaks corresponding to glycoforms with mass shifts of hexose (shown as galactose), *N*‐acetylhexosamine, and *N*‐acetylneuraminic acid (sialic acid) residues are shown (8+ charge state). A split blue/yellow box denotes a residue that can be either *N*‐acetylglucosamine or *N*‐acetylgalactosamine. b) Native mass spectrum of the extracellular domain (ECD) monomer (10+ charge state), containing the CRD plus the neck domain, reveals a similar glycosylation pattern.

**Figure 2:**
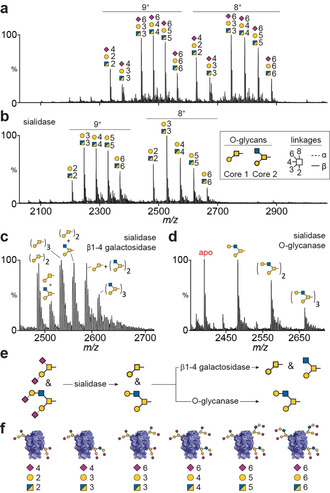
Exo‐ and endoglycosidase digestions of DC‐SIGN CRD. a) Mass spectrum of CRD without enzymatic treatment indicates extensive sialylation of at least six glycoforms in each charge state. b) Sialidase digestion results in five peaks with different hexose and *N*‐acetylhexosamine compositions. c) Sequential digestion with sialidase and β1‐4 galactosidase reveals the presence of both Core 1 and Core 2 O‐glycan structures, (inset in (b)). d) Replacing β1‐4 galactosidase with O‐glycanase, which is specific for Core 1 O‐glycans, results in the formation of the non‐glycosylated CRD (apo peak) and the identification of β1‐4 galactose extensions on Core 2 O‐glycans. e) Schematic of CRD glycan structures generated from exo‐ and endoglycosidase digestions. f) DC‐SIGN CRD glycan macroheterogeneity and microheterogeneity identified from exoglycosidase sequencing.

Since the CRD of DC‐SIGN lacks an Asn‐x‐Ser/Thr sequon, the oligosaccharide PTMs we detected are unlikely to be N‐linked glycans. The numbers of monosaccharides observed by MS are however characteristic of O‐linked glycans, which typically are smaller than N‐glycans, and occur on Ser/Thr amino acids. Covalent attachment of *N*‐acetylgalactosamine is the first step in O‐linked glycan biosynthesis, followed by addition of galactose, *N*‐acetylglucosamine, and sialic acid residues. We cannot infer any structural or occupancy information from observations made in Figure 1 as these values could arise from glycoforms comprised of six core 1 O‐glycans or any number of extended O‐glycan structures.

To overcome this mass degeneracy and probe these glycan structures/microheterogeneity in more detail, we used a limited number of exo‐ and endoglycosidase enzymatic digestions specific for the type and linkage of individual monosaccharides. Exoglycosidases are commonly used in glycomic analyses for trimming individual residues sequentially from the non‐reducing end of oligosaccharides, typically once detached and purified from the underlying protein backbone. Glycan structures (microheterogeneity) become apparent by combining known enzyme specificity with observed peak shifts. Exoglycosidase sequencing has tremendous potential for native MS of glycoproteins by uncovering macroheterogeneity and microheterogeneity information simultaneously.

We applied this combined enzymatic MS approach to DC‐SIGN CRD first using neuraminidase (sialidase) to remove α2‐3,6,8‐linked sialic acids from DC‐SIGN CRD. From the six species identified in Figure 2 a, removal of sialic acids now resulted in five glycoforms with (Hex‐HexNAc)_2–6_ compositions (Figure 2 b). Combining sialidase treatment with a second enzyme (β1‐4 galactosidase), we observed the removal of β1‐4‐linked galactoses yielding six major glycoforms (Figure 2 c) with considerable structural information emerging. The lowermost *m*/*z* peak is CRD with two Core 1 structures (Galβ1‐3GalNAc‐Ser/Thr; yellow circle and square, Figure 2, inset). We are confident in this assignment as other O‐glycan Cores or extended O‐glycan structures can be excluded because they would have an odd value of HexNAc residues, such as an extended Core 3 (Supporting Information, Figure S1). The next peak is 203 Da greater in mass and can be assigned to CRD consisting of one Core 1 and one Core 2 (GlcNAcβ1‐6(Galβ1‐3)GalNAc‐Ser/Thr) glycan. The remaining four peaks in Figure 2 c are CRD glycoforms with (Core 1)_3_, (Core 1)_2_(Core 2)_1_, (Core 1)_1_(Core 2)_2_, and (Core 2)_3_ structures. From this spectrum, we can conclude that the CRD has between two to three Core 1 and Core 2 O‐linked glycans.

With this data alone, the assignment of Core 2 glycans cannot be definitive as these compositions are also equivalent to a Core 3 structure (GlcNAcβ1‐3GalNAc‐Ser/Thr). Core 3 and Core 1 O‐glycans are susceptible to O‐glycanase, so CRDs with Core 1 and Core 3 glycans would result in apo (non‐glycosylated) forms which were detected (Figure 2 d). To confirm our assignment (that is, the absence of Core 3 structures), we treated the CRD with O‐glycanase (following sialidase digestion) to remove unsubstituted Core 1 structures. This resulted in a fraction of apo CRD, which would have previously had two or three Core 1 O‐glycans, with the remaining CRD glycoforms consisting of 1 to 3 extended Core 2 structures (Figure 2 d). This result confirms our glycan assignments in Figure 2 c. Each Core 2 glycan structure contains a single galactose residue (yellow circle), linked β1‐4 to the upper *N*‐acetylglucoasmine residue (blue square). The β1‐4 galactosidase and O‐glycanase digestions (Figure 2 e) therefore confirm both the number of attached O‐glycans (2 to 3) and their specific structures; Core 1 and β1‐4‐galactose‐extended Core 2 with one or two sialic acids linked to penultimate galactoses (Figure 2 f).

Although the presence of O‐glycans on DC‐SIGN has yet to be reported, there is considerable evidence supporting their occurrence. NetOGlyc, a neural network learning algorithm trained by proteome‐wide discovery of O‐glycosylation sites, predicted DC‐SIGN to have three possible O‐glycan sites (Ser383, Ser393, and Thr398).^6^ These sites were also identified using IsoGlyP (http://isoglyp.utep.edu), which allows for selection of specific polypeptide GalNAc transferases (ppGalNAc Ts) in the prediction calculation. Of the 20 known GalNAc Ts, which have different properties that govern peptide O‐glycosylation, HEK293 cells used in this study express GalNAc T1, T2, T3, and T11 (included in our IsoGlyP search). The equivalent search of the DC‐SIGN ECD domain did not change the location or number of O‐glycosylation predictions.

Next, we used ion mobility mass spectrometry (IM‐MS) to probe the stability and structure of CRD glycoforms. Further to mass, IM‐MS reports the rotationally average collision cross section (CCS) of proteins, which relates to their overall size and shape, and consequently can be used to evaluate changes in three‐dimensional structure. We focused on sialidase/O‐glycanase‐treated CRD (herein denoted as CRD^S/O^) due to its homogeneous glycosylation (that is, all glycans on CRD^S/O^ are galactose‐extended Core 2 structures). Besides, the presence of non‐glycosylated proteoforms, this analysis provided a benchmark for interpreting CCS glycoprotein data. This is based on prediction of IM‐MS‐derived CCS values from X‐ray crystal structures, however, to our knowledge only non‐glycosylated proteins have been considered in this way.

Recording the arrival time distributions (ATDs), we observed peak maxima at 4.1 ms (non‐glycosylated), 4.5 ms (one O‐glycan), 4.9 ms (two O‐glycans), and 5.2 ms (three O‐glycans; Figure 3 a,b). The non‐glycosylated CRD^S/O^ CCS (1570 Å^2^) matched the theoretical value (1563 Å^2^) calculated from the CRD X‐ray crystal structure without glycosylation (Figure 3 d).^7^ CCS measurements of CRD^S/O^ with increasing numbers of glycans (1–3) revealed progressively larger structures (Figure 3 c). With the addition of each O‐glycan, the CCS increased in size by approximately 62±2 Å^2^ from the non‐glycosylated CRD^S/O^. Changes in glycoprotein CCSs were therefore consistent and glycan‐specific, pointing to the potential for IM to explore gas‐phase structural variations of glycoproteins.

**Figure 3:**
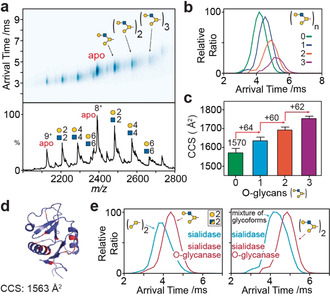
Ion mobility mass spectrometry of CRD glycoforms. a) drift‐plot and MS spectrum of CRD after sialidase and O‐glycanase digestions (CRD^S/O^). Extracted arrival time distributions (b) and collision cross section values (c) of CRD^S/O^ (8^+^ charge state). d) X‐ray crystal structure of non‐glycosylated DC‐SIGN CRD (PDB: 1SL4) with potential O‐glycosylation sites (Ser/Thr residues) highlighted in red. e) ATDs of CRD glycoforms (Hex‐HexNAc)_2_ of identical mass (left plot); one with two Core 1 glycans (blue trace) and one with a single Core 2 glycan (red). ATDs of (Hex‐HexNAc)_4_ equivalent to two Core 2 glycans (red) and mixture of different glycoforms (blue) are illustrated (right).

To explore further the ability of IM to probe these structural variations, we compared CRD of similar mass but different glycosylation. The *m*/*z* of CRD (Gal‐GalNAc)_2_ after sialidase digestion (termed CDR^S^) is the same as CRD^S/O^ with a single galactose‐extended Core 2 O‐glycan. As discussed above, the CDR^S^ (Gal‐GalNAc)_2_ glycoform has two Core 1 structures, meaning two glycan sites are occupied, as opposed to the single extended Core 2 glycan on CRD^S/O^. The IM‐MS arrival times of these two glycoforms were noticeably different (approximately 17 %) revealing distinct CCSs (Figure 3 e). This suggests Core 1 structures induce glycoprotein compaction (that is, lower drift time, 3.8 ms) compared to the single extended Core 2 glycoform (4.1 ms), despite its decrease in mass.

CRDs with two additional monosaccharides (Hex‐HexNAc)_4_ were subsequently examined (Figure 3 e, right). For CDR^S^, a broad ATD was observed, attributed to the presence of multiple glycoforms. By contrast, the ATD for CDR^S/0^, which has two Core 2 glycans, was narrow and more symmetric, implying fewer structures and consistent with a single proteoform. Furthermore, the ATDs increased with the addition of only two monosaccharides (from 3.8 to 4.1 ms for CRD^S^ and 4.5 to 4.9 ms for CRD^S/0^), further highlighting the contribution of glycans to gas‐phase protein structures. Considering the minor contribution of carbohydrate to the overall mass of the glycoprotein, which is 3.6 % for CRDs with (Hex‐HexNAc)_2_ and 7.1 % with (Hex‐HexNAc)_4_ monosaccharides, the difference in ATDs among isomeric glycoforms is apparent. The notion that sugars collapse fully onto the amino acid backbone and play only nominal roles in gas‐phase structures^8^ is therefore inconsistent with our observations.

To assess how these changes in glycosylation affect protein stability, we employed a protein‐unfolding approach using IM to follow CIU, induced by elastic collisions with a neutral gas in the mass spectrometer.^9^, ^10^ During a CIU experiment, the collision voltage is increased incrementally and the protein undergoes unfolding through transition states of different CCSs. The contribution of glycosylation to protein stability can then be compared, similarly as previously described with lipid binding to membrane proteins.^11^ Here, CIU was used to explore stability effects due to glycan microheterogeneity (neutral vs. charged), glycan macroheterogeneity as well as isobaric glycoforms (that is, CRDs with different carbohydrate compositions but equivalent masses).

We examined the CIU profile of four CRD glycoforms, generated by glycosidase digestion (Figure 4 a). A single well‐defined transition was observed for each CIU experiment (Figure 4 b). We noted however that the collision voltage at which glycoform unfolding occurred varied depending on the glycan composition and occupancy. Non‐glycosylated CRDs (*m*/*z* 2128, 19 152 Da) unfolded at the lowest voltage (20 V) while CRDs with a single Core 2 O‐glycan (*m*/*z* 2210, 19 890 Da) unfolded at 23 V. The CRD with the greatest mass (*m*/*z* 2339, 21 051 Da) assigned to two glycans (Hex_2_HexNAc_2_Neu5Ac_4_) had a similar unfolding pattern with a transition at 28 V. The equivalent CRD without sialic acid (two Core 1 glycans; *m*/*z* 2210, 19 890 Da) required significantly higher voltages to induce unfolding (37 V). In summary, the greatest resistance to unfolding (stability) was observed for a CRD with two neutral O‐glycans, followed by a glycoform with two negatively charged glycans, followed by a CRD with a single O‐glycan, which was more stable than the apo protein (Figure 4 c).

**Figure 4:**
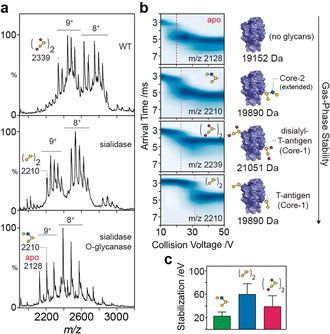
Gas‐phase unfolding of DC‐SIGN CRD glycoforms. a) Native MS spectra of untreated (top), sialidase‐digested (middle), and sialidase‐ and O‐glycanase‐digested (bottom) CRD. The four glycoforms used for unfolding experiments are labelled in each spectrum (9^+^ charge state). b) Corresponding collision‐induced unfolding plots with unfolding transitions (dotted red line) of four CRD glycoforms with varying degrees of glycosylation (right). c) Calculated stability of CRDs with one or two O‐glycans ± sialic acid residues.

These results point to variations in biophysical properties arising from protein glycosylation and a possible role for sialic acids (negatively charged monosaccharide residues) in reducing stability and/or arrangements of glycoproteins in the gas‐phase.

Interestingly, our results indicate that two T antigens on DC‐SIGN contribute to stability more than one Core 2 O‐glycan with the same composition, implying that the number of glycans may affect protein stability more prominently than glycan length. This is in agreement with a previous in silico predictions,^12^ but we could not rule out the possibility of differential stability contributions for each glycosylation sites or that the effect observed here will be true for every glycoprotein. Together these results highlight the potential of native IM‐MS to investigate the stabilization effect of individual glycans, which is challenging using other methodologies.

Given the vast diversity of glycosylation patterns evident for eukaryotic proteins, it is likely that the effects of individual glycans will be highly specific to each individual protein. Nonetheless, native mass spectrometry has great potential to explore the correlations between stability and glycosylation for any protein, as well as uncovering micro‐ and macroheterogenity information in a single experiment. Combining ion mobility and exoglycosidases means analyses can be tailored accordingly. Herein, we showed how this approach not only identified O‐glycosylation of DC‐SIGN but enable us to characterise the extent and effects of glycosylation. These results will lead to a greater understanding of its self‐recognition and potential roles in enhancing viral infections, which in turn inform therapeutic targeting.

## Conflict of interest

H.Y.Y. and I.L. are employees of OMass Therapeutics. W.B.S. is a shareholder and consultant to Refeyn Ltd. J.G. and C.V.R. provide consultancy services to OMass Therapeutics.

## Supporting information

As a service to our authors and readers, this journal provides supporting information supplied by the authors. Such materials are peer reviewed and may be re‐organized for online delivery, but are not copy‐edited or typeset. Technical support issues arising from supporting information (other than missing files) should be addressed to the authors.

Supplementary
