# Supplementary material for: Correlating Glycoforms of DC‐SIGN with Stability Using a Combination of Enzymatic Digestion and Ion Mobility Mass Spectrometry
Source: Angew Chem Int Ed Engl. 2020 Jul 7;59(36):15560–4. doi: 10.1002/anie.202005727 (PMC7496578; doi:10.1002/anie.202005727)
Supplement: Supplementary file 1 — Supplementary [file ANIE-59-15560-s001.pdf]

Angewandte Chemie
Eine Zeitschrift der Gesellschaft Deutscher Chemiker

Supporting Information

Correlating Glycoforms of DC-SIGN with Stability Using a Combination of Enzymatic Digestion and Ion Mobility Mass Spectrometry**

Hsin-Yung Yen, Idlir Liko, Joseph Gault, Di Wu, Weston B. Struwe, and Carol V. Robinson**

anie_202005727_sm_miscellaneous_information.pdf

Author Contributions

Conceptualization: H.Y., I.L., W.B.S. and C.V.R.; Investigation: H.Y., I.L., D.W. and W.B.S.; Formal Analysis: H.Y.; Visualization: H.Y. and W.B.S.; Writing—Original Draft: H.Y. and W.B.S.; Writing—Review & Editing: all authors

Supporting Information

Experimental Section 3
Supplementary Figure 1: Theoretical O-glycans on DC-SIGN CRD. 4
Supplementary Figure 2: Purification of DC-SIGN CRD and ECD. 5
Author Contributions 6

Experimental Section

Construct design and protein purification. The genes of DC-SIGN extracellular domain (ECD; amino residue 66-404) and carbohydrate recognition domain (CRD; amino residue 250-404) were subcloned to pHLsec vector for protein overexpression in HEK293T cells. The condition medium harvested from the cells 48 h post transfection by polyethylenimine, branched average Mw ~25,000Da was harvested for IMAC purification. Sample was loaded into a Histrap column pre-equilibrated with binding buffer (20 mM Tris-HCl, pH7.4, 150mM NaCl and 20 mM imidazole) at flow rate 1ml/min, and protein was eluted with the same buffer containing 500 mM imidazole in a step elution method. The protein fractions were combined together for buffer exchange in 20 mM Tris-HCl, pH7.4, 150mM NaCl to remove imidazole and protein was snap frozen and stored at -80° C. For progressive deglycosylation, purified recombinant DC-SIGN (0.5 ug/ul) was incubated at 37°C for 5hrs with α2-3,6,8 neuraminidase (New England BioLabs) in the ratio of 24 units/1ug purified protein, following the treatment of β1-4 galactosidase or O-glycosidase (New England BioLabs) for another 12hrs at concentration of 5000, 1 units/ul, respectively. The treated samples were applied for MS analysis directly without further storage.

Non-denatured mass spectrometry for DC-SIGN. DC-SIGN CRD was buffer exchanged into 200 mM ammonium acetate, and immediately introduced into a modified Q-Exactive mass spectrometer (prototype Q-Exactive EMR) (Thermo Fisher) according to a previously reported method[12]. Overall a low voltage gradient was applied to transfer optics prior to trapping ions in the higher-energy collisional dissociation (HCD) cell. A low HCD activation voltage (15 V) was used for protein desolvation, in order to avoid protein unfolding and fragmentation of glycan post-translational modifications. For analysis of DC-SIGN ECD, protein sample was pre-treated with 1% acetic acid to dissociate protein complex prior introducing into mass spectrometer. Spectra were acquired with five microscans and averaged with a noise level parameter of 3. Pressure in the HCD cell was increased (measured using UHV pressure ~1.05 x 10^{-9} mbar) to allow better trapping and transmission of protein ions. Data was analysed by using Xcalibur 2.2 SP1.48.

Ion mobility analysis for DC-SIGN. The collisional cross section (CCS) of DC-SGIN was measured using a modified SynaptG2-Si high definition mass spectrometer. Parameters used for analysis were the following: capillary, cone, trap and transfer collision energy were set at 1.3 kV, 50 V, 10 V and 7 V, respectively. The backing pressure was set at 6–8 mbar, and the pressure in the drift cell was set at 4.7 x 10^{-1} bar. The wave velocity and wave height for IMS cell is 500 m/s and 13 V whereas 248 m/s and 8V for Transfer cell. The drift time of four standard proteins (bovine serum albumin, pyruvate kinase, alcohol dehydrogenase, concanavalin A) were acquired under the same instrumental parameters for CCS calculation of DC-SIGN by a home-made software PULSAR[13]. Theoretical CCS of DC-SIGN were calculated using the projection approximation method implemented in MOBCAL and scaled using a scaling factor of 1.14[14]. For protein unfolding experiments, the drift time for each charge state DC-SIGN was obtained under a collisional energy ramp in CID cell with 5V intervals to determine the unfolding pathway. Only non-fragmented glycoforms were analysed. The initial position of each unfolding species was assigned, and its intensity was extracted across all collisional voltages to generate the unfolding model by PULSAR. The stabilization effect (eV) was calculated by the sum of differences of midpoint voltage for each unfolding species in comparison to the non-glycosylated apo CRD, multiplying by individual protein charge state in order to account for charge-dependent factor in protein unfolding.

[12] J. Gault, J. A. C. Donlan, I. Liko, J. T. S. Hopper, K. Gupta, N. G. Housden, W. B. Struwe, M. T. Marty, T. Mize, C. Bechara, et al., *Nat. Methods* **2016**, *13*, 333–336.
[13] T. M. Allison, E. Reading, I. Liko, A. J. Baldwin, A. Laganowsky, C. V. Robinson, *Nat. Commun.* **2015**, *6*, 8551.
[14] Z. Hall, A. Politis, M. F. Bush, L. J. Smith, C. V. Robinson, *J. Am. Chem. Soc.* **2012**, *134*, 3429–3438.

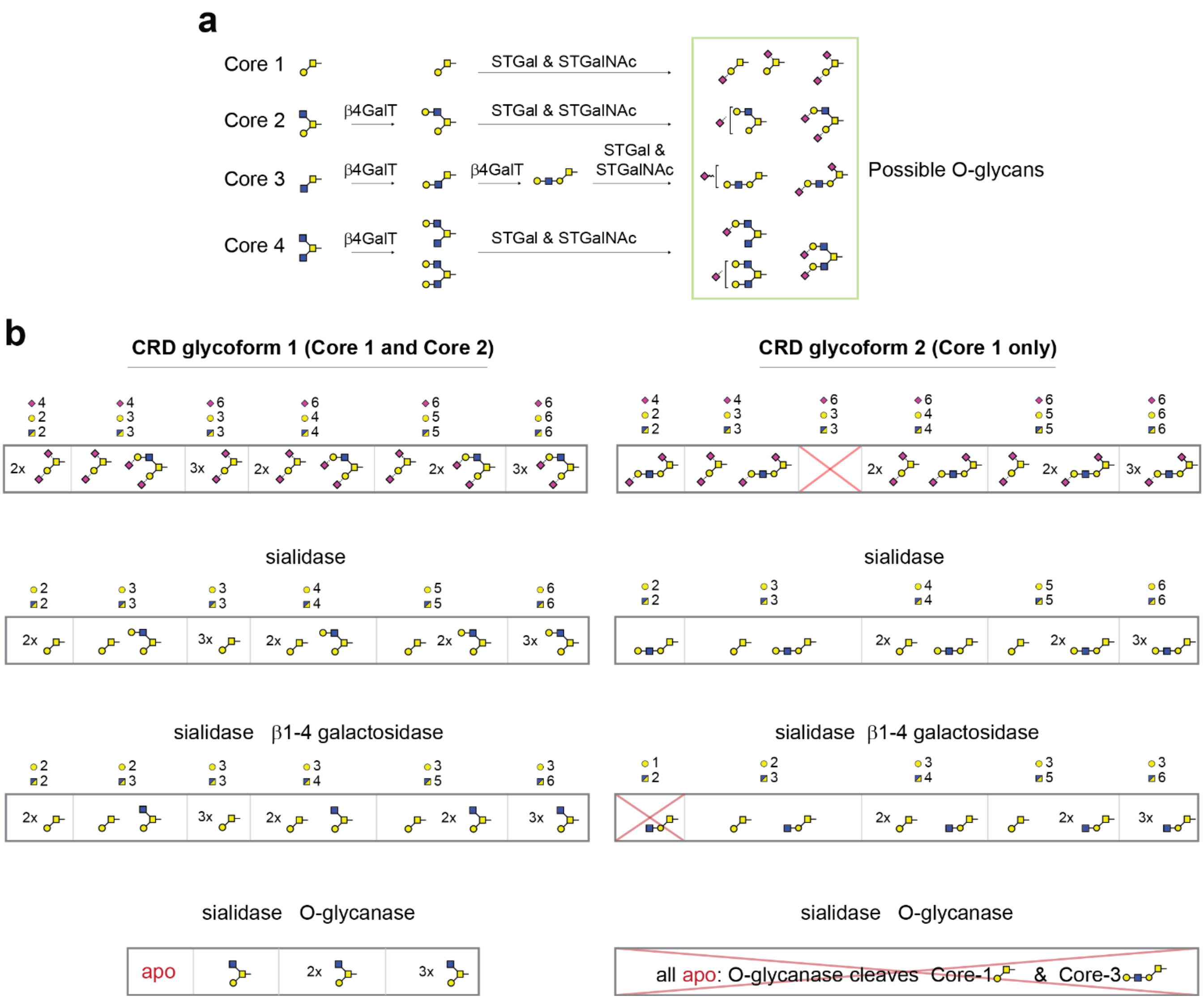

Supplementary Figure 1: Theoretical O-glycans on DC-SIGN CRD. (**a**) Possible O-glycans assembled from Core 1,2,3,4 structures based on addition of β1-4 galactose and α2-3,6 *N*-acetylneuraminic acid. (**b**) Corresponding to exoglycosidase digestions of theoretical O-glycans in (a) show DC-SIGN CRD contain both Core 1 and Core 2 structures.

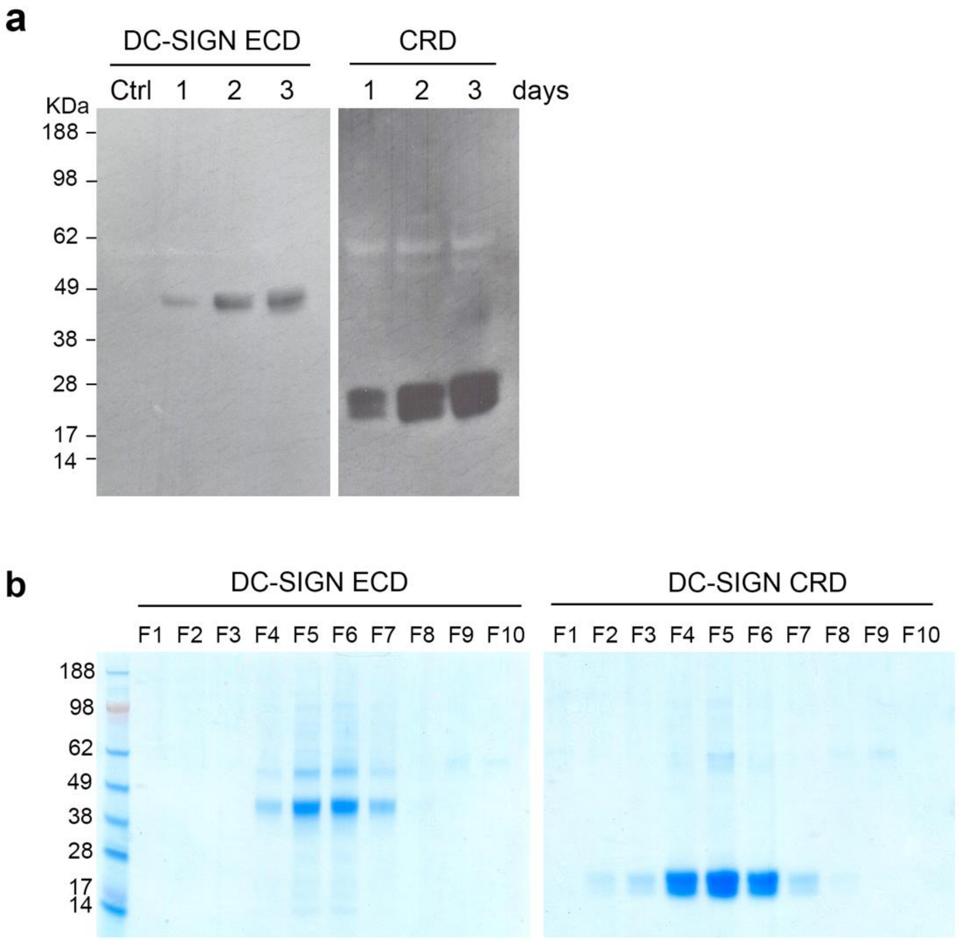

Supplementary Figure 2: Purification of DC-SIGN CRD and ECD. (**a**) Western blotting of condition medium harvested at different time point post transfection by polyethylenimine. (**b**) SDS-PAGE of purified DC-SIGN CRD and ECD from the elution fractions of IMAC.

Author Contributions

Conceptualization: H.Y., I.L., W.B.S. and C.V.R.; Investigation: H.Y., I.L., D.W. J.G., and W.B.S.; Formal Analysis: H.Y.; Visualization: H.Y. and W.B.S.; Writing – Original Draft: H.Y. and W.B.S.; Writing – Review & Editing: all authors
